# Comparison of Fracture Resistance of Endodontically Treated Teeth Restored with FiberSite Postsystem and Glass Fiber, Combined with Different Root Canal Sealers

**DOI:** 10.1155/2021/3818652

**Published:** 2021-10-23

**Authors:** Birgul Eren, Tufan Ozasir, Baris Kandemir, Kamran Gulsahi

**Affiliations:** Department of Endodontics, Faculty of Dentistry, Baskent University, Ankara, Turkey

## Abstract

This study is aimed at evaluating the effects of FiberSite and glass fiber postsystems on the fracture resistance of endodontically restored teeth, when combined with various root canal sealers. Forty human mandibular premolar teeth, each having a single root with anatomically comparable dimension and length, were selected. The teeth in each experimental group (*n* = 10) were instrumented, obturated, and restored with the following procedures: in group 1, AH Plus root canal sealer and a glass fiber post with a composite core; in group 2, AH Plus root canal sealer and a FiberSite postsystem; in group 3, Sure-Seal Root canal sealer and a glass fiber post with a composite core; and in group 4, Sure-Seal Root canal sealer and a FiberSite postsystem. The specimens were placed on a universal test machine. The fracture resistance of all specimens was tested using generic test equipment, and the value of the force in newton (N) during fracture was measured for each root. A statistical analysis was conducted through the Kruskal–Wallis test as well as the post hoc test. The tests showed a significant difference between groups 2 and 3, with group 3 producing load values that were significantly higher than group 2. In contrast, no significant differences were found to exist between the other groups. Regardless of postsystems, groups 3 and 4 showed higher mean fracture values (but no statistically significant differences) than groups 1 and 2.

## 1. Introduction

The teeth with excessive substance loss are more prone to fracture due to caries, trauma, restorative procedures, and endodontic treatments [1]. Prosthetic restorations are generally recommended to restore aesthetics and function in these teeth [2]. To provide conservancy and support to these prosthetic restorations, post and core systems are used that receive support from the root canal [3]. There are many postsystems in different types and materials. For many years, metallic posts, particularly made-to-order cast posts and cores, were the restoration of choice. However, they are favoured less nowadays because of their hardness, high elastic modulus, irregular stress distribution, and susceptibility to corrosion and because they require additional appointments to fit due to the laboratory process [4, 5]. Fiber posts have gained popularity because fiber posts mitigate the possibility of root fracture in endodontically restored teeth due to their dentin-like elasticity modulus [6]. Therefore, they are considered to be a more suitable option than metal posts in most cases [7]. The most popular materials used today as core materials are cast gold, amalgam, dental composite resin, and glass ionomer cement. While amalgam cannot be prepared in the same appointment due to the hardening time, it is similarly disadvantageous to include a second visit in cast postcore systems [8]. Although resin-modified glass ionomer cements can be completed in a single session and have good mechanical properties, they show lower hardness than composite materials and cannot be attached to dentine as securely. Resin composite materials are the preferred core materials as their mechanical properties are close to those of dentine, and they can be prepared and completed in one session [9]. The FiberSite (Mega Dental, Partanna, Italy) is a specialised postcore system that has been recently released which contains a post with an integrated core. The postsystem is composed of fiberglass-reinforced epoxy resin that imitates the core structure of the cut teeth and providing a unified post and core structure [10]. Most of today's root canal sealing methods use a variety of gutta-percha formulations that are filled into the root canal with an insoluble endodontic sealant. Endodontic sealants are designed to fill dental defects and patent accessory canals as well as multiple foramina [11]. There are conflicting results to the effect of root canal sealers on fracture resistance of endodontically treated teeth [12]. However, bioceramic-based sealers are considered a game changer innovation for endodontics [13]. A sealant with an epoxy resin base AH Plus (Dentsply DeTrey GmbH, Konstanz, Germany) is characterised by its nonshrinking nature during polymerisation and its high adaptability to dentin [14, 15]. Bioceramics, which have been popular in recent years, are nano-sized particulate paste. Following the precipitation of calcium and hydroxide ions, a hydroxy apatite layer forms, which provides a chemical connection between the filler and the dentin wall. Sure-Seal Root (Sure Dent Corp., Gyeonggi-do, Korea) is a new root canal filler with a bioceramic base. Sure-Seal Root is a ready-to-use and premixed calcium silicate paste that is easy to use in permanent root canal fillings. It provides exceptional physical properties that allow biocompatibility, improve the sealing of the root canal, and prevent shrinkage during the setting process [16]. In a comprehensive literature review, only one study was found to have investigated the FiberSite postsystem's fracture resistance. For this reason, this study was intended to evaluate the fracture resistance of the FiberSite postcore system and the fiberglass-reinforced postcomposite core systems in the teeth restoratively treated with diverse root canal fillers. The study's null assumption was made that no difference would result between the roots' fracture resistance, which were restored with FiberSite and glass fiber postsystems. The second null hypothesis was to observe better fracture resistance results for roots filled with Sure-Seal Root sealer.

## 2. Materials and Methods

This study was conducted with the financial support of the Baskent University Research Fund (Project No.: D-KA18/14, Ankara, Turkey). G Power 3.1.9. 2 Package program (program written, concept, and design by Franz, Universitat Kiel, Germany; freely available windows application software) was used to determine sample size. It was found appropriate to take at least 40 teeth for the total sample size. The compositions, types, and manufacturers of the materials used in this study are presented in Table 1.

### 2.1. Tooth Selection, Preparation, and Obturation

For this study, 40 single-rooted and single-canal human lower premolar teeth, which were extracted for the purposes of periodontal or orthodontic treatment, were collected. The study excluded two-rooted teeth with immature apices and fractured or decayed roots. The length and dimensions (buccolingual and mesiodistal) of the roots were gauged by means of a digital calliper (Teknikel, Istanbul, Turkey). The teeth of comparable lengths and dimensions at the cementoenamel junction (CEJ) were selected. The selected teeth were then radiographed, both mesiodistally and buccolingually, prior to the operation, to ensure that they had not been previously treated in the root canal or been exposed to resorptions or calcifications. In order to get a uniform root length of 15 ± 1 mm, the crowns were removed at the cementoenamel junction by means of a high-speed diamond disc. A working length of 1.0 mm shorter than the root canal's actual length was defined by using the k-file (size # 10, VDW, Munich, Germany). Roots were subjected to an instrumented process with Mtwo rotary files up to # 40/0.04 taper (VDW, Munich, Germany). The root canals were then irrigated using 1 mL of 2.5% NaOCl (CanalPro; Coltene, Altstätten, Switzerland) between each instrument. A final irrigation was performed on the canals with 2 mL of 2.5% NaOCl for 1 minute, 2 mL of 17% ethylenediamine tetra acetic acid (EDTA) (CanalPro; Coltene) for 1 minute, and 5 mL of distilled water, respectively, after which the root canals were dried using paper points. The teeth were then randomly grouped, creating two major groups (*n* = 20/each) according to the type of root canal sealers. A total of 20 teeth were filled with AH Plus root canal filler combined with a single ISO size # 40, 0.04 tapered gutta-percha master cone. AH Plus sealer was blended as prescribed by the manufacturer, followed by the master gutta-percha cone lightly coated with the AH Plus sealer and its subsequent insertion inside the canal up to the working length indicated, and then, the cone was cut at the orifice level by using a warm excavator. Following this procedure, a vertical compaction was ultimately performed using a size 11 plugger to a depth of about 1 mm into the canal opening. The group of 20 teeth was then divided into two subgroups: groups 1 and 2 (*n* = 10/each). The remaining 20 teeth were filled with Sure-Seal Root canal sealer combined with a single ISO-sized # 40, 0.04 tapered gutta-percha master cone in the same manner as groups 1 and 2. These specimens were also divided into two subgroups: groups 3 and 4 (*n* = 10/each). The coronal access of roots was filled with a glass ionomer cement (3M Vitremer, 3M Dental products, USA). To ensure that the root canal sealer set thoroughly, all samples were then kept at a temperature of 37°C and a humidity of 100% for 14 days.

### 2.2. Postpreparation

Both postsystems were provided with postspaces of 11 mm depth from the cement-enamel junction, created in all samples by means of their respective drills provided by the products. A 4 mm long apical root filling was left in the canal. Following instrumentation, the postspaces were irrigated with 2 mL of 17% EDTA for 2 min, to remove the smear layer, and were then rinsed with 5 mL of distilled water and dried with paper points. The samples were restored with the following procedures: in group 1, glass fiber post with a composite core (AH Plus); in group 2, FiberSite postsystem (AH Plus); in group 3, glass fiber post with a composite core (Sure-Seal Root); and in group 4, FiberSite postsystem (Sure-Seal Root).

The prepared postspace was treated with Clearfil™ S3 Bond Plus (Kuraray Medical Inc., Tokyo, Japan) for 10 seconds using a microbrush, which was then air-dried and cured under light for 10 seconds (Elipar S10; 3M ESPE). Then, the postspace and the postsurface were filled with Clearfil™ DC Core Plus (Kuraray Medical Inc., Tokyo, Japan).

All posts (glass fiber posts (groups 1 and 3) and FiberSite posts (groups 2 and 4)) were then gently inserted into the postspaces by exerting pressure with a finger. Any excess resin cement was brushed off using a microbrush. The resin was set in a position perpendicular to the post and was cured using light-curing equipment for 40 seconds (Elipar S10; 3M ESPE) and then for a further 6 minutes without light-curing. The core structures for fiber postgroups were made using an incremental technique with resin composite material. FiberSite postapplicators were removed and light cured for it contains built-in composite core. Subsequently, all samples were kept in an incubator at a temperature of 37°C and a humidity of 100% for 24 hours to finalize the chemical curing process.

### 2.3. Preparation for the Fracture Resistance Test

The surface of each root was coated with modelling wax and then embedded into a cylindrical plastic tube (5 cm height and 3 cm diameter) using a self-curing acrylic resin (Imicryl, Konya, Turkey), allowing a 1.5 mm exposure of each root. Then, the modelling wax was removed, and polyvinyl siloxane light body (Zhermack, 45021 Badia Polesine, Rovigo, Italy) was injected into the space formed to simulate the periodontal ligament, after which the roots were positioned back inside the blocks. Following the seating of the impression material, any excess material was removed using a scalpel. By means of a universal tester (Lloyd LRX, Lloyd Instruments, Fareham, UK) at a steady speed (1.0 mm/min) and at an angle of 135° to the long axis of the sample centrally using 6 mm round tip, the fracture test was performed until a fracture was achieved. For each root, the force required to fracture was measured in newton (N).

### 2.4. Statistical Analysis

A statistical analysis was conducted on the data using the Kruskal–Wallis test, as well as the post hoc Scheffe test, to find out the differences that may exist between the groups, using the SPSS 20.0 (IBM-SPSS Inc., Chicago, IL, USA) software program. The statistical significance level was designated as *p* < 0.05.

## 3. Results

Table 2 shows the mean values ± standard deviations, in addition to the median, minimum, and maximum values of the fracture resistance for each experimental group. The results of multiple comparison tests between all groups are demonstrated in Table 3. For the experimental groups 1, 2, 3, and 4, the mean values were found to be 281.9 N, 254.5 N, 328.0 N, and 305.0 N, respectively, which revealed a statistically significant difference in the comparison made between group 3 and group 2 (*p* < 0.05). Group 3 (glass fiber with a composite core and Sure-Seal Root) had significantly higher load values compared to group 2 (FiberSite and AH Plus). In contrast, although no significant differences were found to exist in terms of fracture resistance between groups 1, 3, and 4, the roots sealed using Sure-Seal Root canal sealant in groups 3 and 4 yielded the highest mean values in fracture, being 328.0 N and 305.0 N, respectively.

## 4. Discussion

Since the artificial teeth do not simulate dentine, this study used the natural teeth to achieve clinical conditions [17]. The natural teeth differ in mechanical properties and size [18]. To achieve a standardized sample, the study used roots of similar size, length, and dimensions. In this study, periodontal membrane application was found appropriate to imitate the natural tooth structure. The polyvinyl siloxane (impression material) was used in this study because its modulus of elasticity is close to the periodontal ligament [19]. Various postsystems were evaluated for fracture resistance. The fiber-based postsystems are recommended over metal posts to prevent irreparable root fractures [6]. The FiberSite postsystem used in this study is a new system, in which the post and core materials are designed as a single unit. This product consists of a fiberglass-reinforced post and glass fiber reinforced epoxy resin. The concept of monoblock restoration is that the post and core are formed with solid interconnected materials. Thus, the restoration becomes a single structure that reinforces the tooth structure [20]. The results produced in the present in vitro study (Table 3) showed that the teeth in group 3 (glass fiber with a composite core and Sure-Seal Root) had significantly higher fracture values compared to group 2 (FiberSite and AH Plus) (*p* < 0.05). This result is also consistent with the findings of an earlier study, which reported that the fracture resistance of the Relyx fiber post was significantly higher than that of the FiberSite post and can be explained by the design of the posts [21]. Certain fiber posts feature grooves on the posts that are designed to ensure that they are retained in the coronary segments and which can adversely affect the fracture resistance of the posts. The glass fiber posts have no grooves, while the FiberSite posts feature grooves and a double-stage taper design. Milot and Stein [22] revealed that the surface of a core segment abutting the dentin had a more substantial effect on the fracture resistance of the teeth subjected to root canal treatment (*p* < 0.05) than the design of the post. This study showed that the fracture values of the group 2 (FiberSite and AH Plus) were significantly lower than the group 3 (glass fiber with a composite core and Sure-Seal Root). This result could be attributed to the FiberSite's core design. It is probable that the fracture resistance of the FiberSite postspecimens were decreased by the design of the core of the FiberSite post where the post abuts the coronal section of the root, thereby rejecting the null assumption. It was reported by Schwartz et al. [23] that the success of the teeth placed in the canal varies according to the type of post, filling material, and sealer selected. In this study, AH Plus (with a resin base) and Sure-Seal Root (with a bioceramic base) root canal filling materials were used. It has been shown by numerous studies that AH Plus root canal sealer has a greater strength of adhering to the root canal dentin and might enhance the strength of endodontically treated teeth against root fracture in comparison to other root canal fillers [24–26]. Sure-Seal Root, a new root canal sealer with a bioceramic base, is preferred for its qualities such as superior sealing and easy spreading to the root canal system and accessory canals, due to its low surface tension and high hydrophilic structure [16]. The results from this study showed no differences in terms of fracture strength between groups 1, 3, and 4, whereas roots filled with Sure-Seal Root canal sealer (as group 3 and 4) yielded higher, albeit insignificantly different, fracture values compared to AH Plus-filled roots (as in group 1). The fracture resistance of the teeth sealed with different root canal sealants has been evaluated in numerous studies [27–29]. Consistent with this study, Topçuoğlu et al. [27] indicated that no differences were found to exist in terms of fracture resistance between the roots sealed with AH Plus and Endosequence BC (bioceramic sealer). On the contrary, Mandava et al. [28] revealed a higher fracture resistance in roots sealed using AH Plus sealant compared to the roots filled with Meta SEAL and MTA Fillapex (bioceramic sealer). This difference may stem from the design of the study, for example, the type of bioceramic-based sealer used and the sample size. In order to verify the safe use of the FiberSite postsystem, there is a need for further extended clinical studies.

The absence of fractographical evaluation is a limitation of this study. Fractographical evaluation has been performed in some studies [30–32] by using the combination of stereo and scanning electron microscopy techniques. The stereomicroscope allows to see the area as a whole where the fracture occurs, while the SEM analysis gives detailed information about the fracture especially if it is a cohesive fracture that occurred, showing the fractographic markers, but in this method, adhesive fractures are not visible [32].

## 5. Conclusions

Within the limitations of this study, Sure-Seal, while not being statistically significant, showed better fracture resistance values than AH Plus. FiberSite postsystem showed poorer performance than glass fiber posts especially when used in conjunction with AH Plus sealer.

## Figures and Tables

**Table 1:** The compositions, types, and manufacturers of the materials.

Trade	Type	Chemical composition	Manufacturer
FiberSite	Glass fiber, postcore system	Glass fiber, epoxy resin matrix	Mega Dental, Partanna, Italy
Cytec Blanco	Glass fiber post	Glass fiber, epoxy resin matrix	Hahnenkratt, Konigsbach-Stein, Germany
AH Plus	Epoxy-resin-based root canal sealer	Epoxy resins, zirconium oxide, iron oxide, calcium tungstate, silicone oil	Dentsply DeTrey GmbH, Konstanz, Germany
Sure-Seal Root	Bioceramic-based root canal sealer	Zirconium, bioactive glass, glass ceramic, radiotherapy glass, composites of hydroxyapatite, resorbable calcium phosphate	Sure Dent Corp., Gyeonggi-do, Korea

**Table 2:** Mean ± standard deviation, median, minimum, and maximum values of fracture resistance for each group (in newton).

Groups	*n*	Mean ± SD	Median	Min	Max
Glass fiber + AH Plus	10	281.9 ± 66.5	295.2	194.8	399.2
FiberSite + AH Plus	10	254.5 ± 69.1	245.3	177.0	424.0
Glass fiber + Sure-Seal	10	328.0 ± 89.9	331.4	148.0	476.6
FiberSite + Sure-Seal	10	305.0 ± 44.6	284.9	262.7	396.9

SD: standard deviation.

**Table 3:** Statistical multiple comparison of experimental groups.

Compared groups	*p* value
Group 1/group 2	*p* = 1.00
Group 1/group 3	*p* = 0.942
Group 1/group 4	*p* = 1.00
Group 2/group 3	*p* = 0.037^∗^
Group 2/group 4	*p* = 0.28
Group 3/group 4	*p* = 1.00

^∗^Differences between groups identified were statistically significant (*p* < 0.05).

## Data Availability

The research article data used to support the findings of this study are included within the article.

## References

[1] Gutmann J. L. (1992). The dentin-root complex: anatomic and biologic considerations in restoring endodontically treated teeth. *The Journal of Prosthetic Dentistry*.

[2] Reeh E. S., Messer H. H., Douglas W. H. (1989). Reduction in tooth stiffness as a result of endodontic and restorative procedures. *Journal of Endodontia*.

[3] Zhou L., Wang Q. (2013). Comparison of Fracture Resistance between Cast Posts and Fiber Posts: A Meta- analysis of Literature. *Journal of Endodontia*.

[4] Manjunath P., Sujatha I., Jayalakshmi K. B., Latha P. (2017). Comparison of fracture resistance of endodontically treated teeth restored with two different fiber posts. *International Journal of Applied Dental Sciences*.

[5] Schwartz R. S., Robbins J. W. (2004). Post placement and restoration of endodontically treated teeth: a literature review. *Journal of Endodontia*.

[6] Bateman G., Ricketts D. N. J., Saunders W. P. (2003). Fibre-based post systems: a review. *British Dental Journal*.

[7] Cagidiaco M. C., Goracci C., Garcia-Godoy F., Ferrari M. (2008). Clinical studies of fiber posts: a literature review. *The International Journal of Prosthodontics*.

[8] Sahmali S. M., Saygili G. (2020). Compressive shear strength of core materials and restoring techniques. *The International Journal of Periodontics & Restorative Dentistry*.

[9] Subash D., Shoba K., Aman S., Indu Bharkavi S. K., Nimmi V., Abhilash R. (2017). Fracture resistance of endodontically treated teeth restored with biodentine, resin modified GIC and hybrid composite resin as a core material. *Journal of Clinical and Diagnostic Research*.

[10] MegaDental Brochure products. https://www.fibersitepost.com/depliantsgrande.pdf.

[11] Gulsahi K., Cehreli Z. C., Kuraner T., Dagli F. T. (2007). Sealer area associated with cold lateral condensation of gutta-percha and warm coated carrier filling systems in canals prepared with various rotary NiTi systems. *International Endodontic Journal*.

[12] Almohaimede A., Almanie D., Alaathy S., Almadi E. (2020). Fracture resistance of roots filled with bio-ceramic and epoxy resin-based sealers: <i>In Vitro</i> study. *European Endodontic Journal*.

[13] Koch K. (2009). Bioceramic technology—a game changer in endodontic obturation. *NJAGD Wisdom*.

[14] Grossman L. I. (1976). Physical properties of root canal cements. *Journal of Endodontia*.

[15] McComb D., Smith D. C. (1976). Comparison of physical properties of polycarboxylate-based and conventional root canal sealers. *Journal of Endodontia*.

[16] Saghiri M. A., Karamifar K., Nath D., Gutmann J. L., Sheibani N. (2021). A novel polyurethane expandable root canal sealer. *Journal of Endodontia*.

[17] Mendoza D. B., Eakle W. S., Kahl E. A., Ho R. (1997). Root reinforcement with a resin-bonded preformed post. *The Journal of Prosthetic Dentistry*.

[18] Sidoli G. E., King P. A., Setchell D. J. (1997). An in vitro evaluation of a carbon fiber-based post and core system. *The Journal of Prosthetic Dentistry*.

[19] Ko C. C., Chu C. S., Chung K. H., Lee M. C. (1992). Effects of posts on dentin stress distribution in pulpless teeth. *The Journal of Prosthetic Dentistry*.

[20] Khatavkar R. A., Hegde V. S. (2010). The weak link in endodontics: gutta-percha—a need for change. *World Journal of Dentistry*.

[21] Özyürek T., Topkara C., Koçak İ., Yılmaz K., Gündoğar M., Uslu G. (2020). Fracture strength of endodontically treated teeth restored with different fiber post and core systems. *Odontology*.

[22] Milot P., Stein R. S. (1992). Root fracture in endodontically treated teeth related to post selection and crown design. *The Journal of Prosthetic Dentistry*.

[23] Schwartz R. S., Murchison D. F., Walker W. A. (1998). Effects of eugenol and noneugenol endodontic sealer cements on post retention. *Journal of Endodontia*.

[24] Nagas E., Altundasar E., Serper A. (2009). The effect of master point taper on bond strength and apical sealing ability of different root canal sealers. *Oral Surgery, Oral Medicine, Oral Pathology and Oral Radiology Endodontology*.

[25] Fisher M. A., Berzins D. W., Bahcall J. K. (2007). An in vitro comparison of bond strength of various obturation materials to root canal dentin using a push-out test design. *Journal of Endodontia*.

[26] Ersev H., Yilmaz B., Pehlivanoǧlu E., Özcan-Çalişkan E., Erişen F. R. (2012). Resistance to vertical root fracture of endodontically treated teeth with MetaSEAL. *Journal of Endodontia*.

[27] Topçuoǧlu H. S., Tuncay Ö., Karataş E., Arslan H., Yeter K. (2013). _In Vitro_ Fracture Resistance of Roots Obturated with Epoxy Resin-based, Mineral Trioxide Aggregate-based, and Bioceramic Root Canal Sealers. *Journal of Endodontia*.

[28] Mandava J., Chang P. C., Roopesh B., Faruddin M. G., Anupreeta A., Uma C. (2014). Comparative evaluation of fracture resistance of root dentin to resin sealers and a MTA sealer: an in vitro study. *Journal of Conservative Dentistry*.

[29] Celikten B., Uzuntas C. F., Gulsahi K. (2015). Resistance to fracture of dental roots obturated with different materials. *BioMed Research International*.

[30] Rocca G. T., Saratti C. M., Cattani-Lorente M., Feilzer A. J., Scherrer S., Krejci I. (2015). The effect of a fiber reinforced cavity configuration on load bearing capacity and failure mode of endodontically treated molars restored with CAD/CAM resin composite overlay restorations. *Journal of Dentistry*.

[31] Comba A., Baldi A., Saratti C. M. (2021). Could different direct restoration techniques affect interfacial gap and fracture resistance of endodontically treated anterior teeth?. *Clinical Oral Investigations*.

[32] Scotti N., Forniglia A., Michelotto Tempesta R. (2016). Effects of fiber-glass-reinforced composite restorations on fracture resistance and failure mode of endodontically treated molars. *Journal of Dentistry*.

